# Supplementary material for: Are carbon emissions trading and green financial instruments synergistic? -Comprehensive quantitative research based on content analysis
Source: PLoS One. 2024 Mar 7;19(3):e0298601. doi: 10.1371/journal.pone.0298601 (PMC10919601; doi:10.1371/journal.pone.0298601)
Supplement: S1 Data — (ZIP) [file pone.0298601.s001.zip › Data/地级市Z_F工作报告.zip!/║ú─╧/║ú┐┌╩╨/└┤╫╘╣1⁄2╓┌║┼í╛┬φ┐╦╩2╛▌═°í┐_2003.docx]

各位代表：

　　 现在，我代表市人民政府，向大会作工作报告，请予审议，并请各位政协委员和其他列席人员提出意见。

　　 一、过去五年的工作回顾

　　 五年来，在省委、省政府和市委的正确领导下，本届政府带领全市人民高举邓小平理论伟大旗帜，认真实践“三个代表”重要思想，团结奋进，开拓创新，胜利完成市十二届人大一次会议确定的各项任务，“九五”计划按期完成，“十五”计划开局良好。

　　国民经济走出低谷，经济运行步入快速健康发展轨道 。2002年，完成国内生产总值211.04亿元，比1997年增长62.1%，年均增长10.1%，高出全国2.4个百分点。经济运行质量和效益明显提高，财政收入32.5亿元，比1997年增长78.5%，年均增长12.3%；五年财政总支出79.24亿元，比上五年增加21.99亿元；城镇居民人均可支配收入8004元，农民人均纯收入3103元(新口径)，年均分别增长4%和5%，人民生活总体上达到小康水平。

“两地一中心”发展战略全面实施，优势产业发展迅猛 。2002年，完成工业总产值161.38亿元，比1997年增长1.2倍，年均增长17%；接待过夜旅游人数391.4万人次，旅游总收入33.75亿元，分别比1997年增长33.1%和63.1%；社会消费品零售总额101.96亿元，比1997年增长50.3%；农业总产值33.89亿元，比1997年增长80.3%。高科技园区和新兴工业区建设步伐加快，海汽、海宇、兴业聚酯等企业生产规模不断扩大，海富制药、清华显示器等一批新的工业项目发展势头强劲；工业总体素质明显提高，全市高新技术产品产值占工业总产值31%，全省高新技术产业基地初步形成。旅游精品不断涌现，“琼州海峡旅游度假区”、“海口旅游圈”初具规模，全国热带滨海旅游胜地构架初步形成。仓储、配送、连锁、专卖等现代物流业方兴未艾，人流、物流、资金流、信息流的服务功能进一步增强，区域性商贸中心建设明显加快。特色优质高效农业进一步发展，农业基础地位不断加强。

　　结构调整成绩显著，经济泡沫基本消除 。所有制结构进一步调整和完善，各种所有制经济竞相发展，个体、私营等非公有制经济在增加就业、活跃市场、扩大出口等方面发挥了重要作用。产业结构日趋合理，三次产业由1997年的10.7:27.9:61.4调整为2002年的11.2:30.9:57.9。处置积压房地产和化解金融风险取得突破性进展。44.3%的空置商品房顺利消化，闲置建设用地基本处理完毕，近一半停缓建工程复工建设；金融秩序明显好转，对经济建设的支持力度不断加强。

　　城市建设步伐加快，创建工作成绩突出 。五年累计完成市政设施投资近30亿元。改造国兴大道、秀英大道、海府路、海秀东路等城市干道，完成南渡江防洪堤、海甸溪防潮堤等防洪防潮工程，建成白沙门污水处理厂、颜春岭垃圾处理场等市政工程，建设世纪大桥、滨海立交桥、西海岸带状公园、美舍河整治等重点骨干项目，城市基础设施日趋完善。认真开展环境综合整治，建成区绿化覆盖率达43.4%，空气质量始终保持全国最优。先后荣获中国优秀旅游城市、全国城市环境综合整治优秀城市、国家环境保护模范城市、全国造林绿化十佳城市、全国卫生先进城市和国家园林城市等10多个荣誉称号，塑造了城市新形象。

　　各项社会事业全面发展，城市文明程度明显提高 。“科教兴市”深入实施，高新技术广泛应用，科技成果不断涌现；学校建设得到加强，“两基”达标成果不断提高，素质教育成效明显。实施“文化精品”战略，涌现出《达达瑟》、《赤色小子》等一批享誉全国的文艺作品。体育、卫生、计划生育、广播电视等各项社会事业全面进步。双拥工作成绩突出，连续四次荣获“全国双拥模范城”。不断拓宽就业再就业渠道，实现就业再就业6万多人次，连续五年城市登记失业率控制在3.5%以下。社会保障体系进一步完善，“两个确保”率达100%；加强“低保”工作，五年财政共安排资金3000万元，全市28.9万困难群众享受最低生活保障，基本做到应保尽保。建立市特困居民援助中心，全力扶助社会弱势群体，特困居民的“六难”问题得到初步解决。积极实施“打、防、控、管、建”相结合的社会治安综合治理，荣获全国社会治安综合治理优秀单位，全市政治安定，社会稳定。

　　改革开放稳步推进，城市活力不断增强 。以优化资本结构为突破口的国有企业改革取得重大突破。43家市属国有工业企业完成改制，66家国有商贸企业全部退出国有序列，海药、海德、金盘等国有控股公司实现资产重组，消除30多亿元的不良债务，新增投入和盘活国有资产20多亿元。大力整顿和规范市场经济秩序，市场环境和消费环境逐步改善。招商引资取得较大进展，一批外资项目相继落户，五年实际利用外资18.62亿美元，进出口总额38.01亿美元，对外开放形成新的局面。

　　政府自身建设不断加强，发展软环境进一步改善 。积极推进政府机构改革和行政审批制度改革，加强法规建设，推进依法行政，强化民主监督，不断深化“三制”，运用“双评”机制，全面开展现代文明机关创建活动，机关工作作风明显改进，办事效率和服务水平明显提高。大力强化“善待企业、善待游客、善待市民”的理念，建立重点项目服务中心和投资环境监督中心，政务环境进一步优化。全面落实廉政建设责任制，积极开展行业不正之风的专项治理，强化行政执法监察，加大违纪违法案件特别是大案要案的查办力度，反腐倡廉工作取得新的成效。

　　 五年的成就令人鼓舞，五年的经验催人奋进。五年来，我们始终坚持高举邓小平理论伟大旗帜，全面贯彻党的十五大、十六大和省四次党代会精神，把发展作为第一要务，抢抓发展机遇，不断开创改革开放和现代化建设新局面；始终坚持解放思想、实事求是，形成符合海口实际的发展思路，“两地一中心”发展战略不断完善；始终坚持以“三个代表”重要思想统揽全局，努力提高城乡人民生活水平，最大限度地调动广大人民群众的积极性、主动性和创造性；始终坚持“两手抓、两手都要硬”，促进两个文明协调发展；始终坚持依法治市与以德治市相结合，妥善处理好改革、发展、稳定的关系，有力地保证全市改革开放和经济建设的顺利进行。

　　 各位代表，过去的五年，我们走过了不平凡的历程，取得了来之不易的成就。这是省委、省政府正确领导的结果，是历届市委市政府开拓创新、团结拼搏的结果，是全市党政军民和社会各界共同努力的结果。在此，我代表市政府向所有为海口改革发展作出贡献的同志们，向所有关心支持海口建设的投资者和朋友们，表示衷心的感谢并致以崇高的敬意！

　　 在充分肯定成绩的同时，我们也清醒地认识到亟待解决的主要矛盾和问题：经济总量不够大，结构不尽合理，支柱产业不够强，财源基础脆弱，吃饭财政的局面尚未得到根本改变；城市规划、建设、管理仍需进一步加强；社会有效需求不足，物价持续走低，市场供大于求的格局还没有明显改变；就业和再就业压力增大，城乡居民收入增长缓慢，一些群众生活仍比较困难；政府职能转变与新形势、新任务的要求还不完全适应，软环境建设还不容乐观；影响稳定的因素还不同程度存在，等等。对此，我们要继续采取有力措施，认真加以解决。

二、今后的奋斗目标和总体要求

　　 九州迎盛世，海口望未来。未来20年是我市加快发展的重要战略机遇期。按照党的十六大精神和市第十次党代会的部署，我市全面建设小康社会的总体发展目标是：到2010年，人均国内生产总值比2000年翻一番，达到4000美元，综合经济实力迈上新台阶，中心城市辐射带动作用明显增强，提前10年率先基本实现全面建成小康社会的奋斗目标。到2020年，人均国内生产总值再翻一番，达到8000美元，在全省率先基本实现现代化。把海口建成我国一流生态环境，一流人居环境，一流创业环境的城市；建成环北部湾的重要经济城市；建成文明富裕、遍地葱绿、鲜花盛开的四季花园城市；建成具有热带风光滨海特色的绿色国际性城市。

　　 实现上述目标，我们具有良好的基础和必要的条件。历届政府为我市经济加快发展打下了良好的基础；行政区划的调整为我市加快发展提供了丰富的资源；固定资产投资不断增大为我市经济加快发展提供了重要保障。

未来五年是我市实现“两个翻番，两个率先”的关键时期。我们的总体要求是：

　　 必须在规模、结构、质量、效益有机结合的基础上，扩大经济总量，全面加快经济发展。到2007年，全市综合经济实力明显增强，人均国内生产总值达到3500美元；经济质量和效益明显提高，经济结构明显优化，三次产业比例更趋合理，城镇化水平达到60%以上，城乡差别进一步缩小；全市固定资产投资累计达700亿元以上，在全省发展中的龙头地位更加明显。

　　 必须立足我省建设中华民族四季花园和全国人民度假村，着眼海口的长远发展，做好城市总体规划。按照“突出沿海，开发沿江，提升中心，拓展两翼，带动腹地”的要求，进一步明确城市定位和发展方向，完善基础配套，提升城市功能，优化生态环境，美化城市景观，提高城市品位。

　　 必须以提高市民素质为重点，以提高人民的生活水平和质量为目标，加强精神文明建设，促进各项社会事业健康协调发展。完善国民教育、科技、医疗卫生三大体系，巩固“两基”成果，发展壮大教育产业，逐步形成全民学习、终身学习的学习型城市；广开就业门路，促进城乡就业和再就业，实现城镇登记失业率控制在3.5%以下；完善城镇社会保障体系；使城镇居民可支配收入、农民纯收入达到全国先进水平。

　　 必须在坚持党的领导下，逐步完善社会主义民主政治与法制体系，推进依法行政。发展社区民主，扩大企业民主，推进村民自治，发挥社会多主体多层次多形式的监督作用，维护广大人民依法享有民主选举、民主决策、民主管理、民主监督的权力。健全城乡管理各种法规，坚持把党的领导、人民当家作主和依法行政有机统一起来，努力推进依法治市。

　　 小康建设，重在开局。2003年经济社会发展的预期目标是：

　　 国内生产总值增长12%，财政收入增长12%，固定资产投资增长15%，社会消费品零售总额增长11%，城镇居民人均可支配收入增长8%，农民人均纯收入增长6%，人口自然增长率控制在7.2‰以内。

　　 实现上述目标，必须高举邓小平理论伟大旗帜，全面贯彻“三个代表”重要思想，认真落实党的十六大、省第四次党代会和市第十次党代会精神，抓住行政区划调整的契机，坚持把发展作为第一要务，不断推进城市化战略、优势产业战略、开放带动战略、绿色发展战略、科教兴市战略。加快城市建设，美化生态环境；优化经济结构，夯实产业基础；深化改革开放，增创体制优势，与时俱进，开拓创新，努力做大做强做精做美新海口。

三、搞好总体规划，加快基础设施建设，全面提高城市建设水平

　　 环境是城市的绿色血脉，城市是凝固的文化诗篇。我们要紧紧抓住优化城市生态环境和丰富城市文化内涵这两个重点，按照“高起点规划、高标准建设、高效能管理”的要求，抓住新机遇，运用新思路，采取新举措，建设新海口。

　　 城市规划是城市建设的龙头。紧紧抓住新海口城市功能定位、城市内涵和产业布局，本着“以人为本，生态优先”的原则，高起点编制城市总体规划、分区规划和修建性详规，科学编制产业发展规划，精心编制交通、电讯、园林、环境、灯光等专业规划，形成特色鲜明、个性突出的特色和风格。规划编制既要尊重专家的意见，又要广泛采纳市民的建议，提高规划的科学性。加强规划宣传和教育，增强全体市民的规划意识和执行规划的自觉性。强化规划执法和监督，确保规划的权威性和严肃性。

　　 加强城市基础设施建设是提升城市综合竞争力的重要保证。重点是积极筹措建设资金，加强道路系统建设，拉开城市的骨架。贯通大环市路，南部建设海文公路和西线公路的连接大道，东部建设滨江西路，北部改造扩建滨海大道，西部延长滨海西延线。疏通市内道路网络，改造迎宾大道、南海大道、滨海西路等主干道，打通国兴大道、和平南路、新港路、海甸四西路等断头路，拓宽美化金贸大道、和平大道等十字路。按照港口的功能定位，抓紧制定马村港、秀英港、新港和北港的建设规划，做好“四港分离”的前期准备，努力形成相互协调、功能完善的城市水路交通体系。调整优化公共服务设施，进一步完善城市“五网”等公用设施和文化、教育、卫生、体育、社会福利等公共服务体系。加大处置积压房地产力度，创新资产置换办法，创新代为处置方式，盘活积压房地产。

　　 提高城市管理水平是城市健康有序发展的重要保障。完善城市管理体制，推进城市管理重心下移，调动基层和广大市民管理城市的积极性，实现人民城市人民管的良好局面。稳步推进相对集中处罚权工作，开展城市综合执法，提高执法效率。整顿市内交通秩序，打击非法营运、野蛮营运，切实解决交通秩序混乱的局面。加强城市管理队伍建设，建立一支政治强、业务精、作风好的执法队伍，促进城市管理上新的台阶。

　　 建设绿色生态城市是实现可持续发展的重要前提。以建设花园城市为目标，打生态环境牌，走绿色发展路，实现市区园林生态化、城市周边森林化、农村田园化。加快编制城市绿地生态系统规划、园林建设规划、水体系统规划、城市亮化规划。要多种树，还绿于民。建设全市五大出口生态森林体系和花卉基地，建设城边、村边、路边“三边”绿化带，对市区主干道实施园林绿化改造，最大限度地提高城区的绿地率，2007年城市绿化覆盖率达到45%以上。切实保护地下水资源，坚决治理污水，加强南渡江、海岸线和东西湖、红城湖、美舍河等整治，使江河湖海成为城市最亮丽的风景。要多整治，还海于民。加快建设东西海岸、南渡江生态景观长廊、市民中心广场、世纪广场等城市精品景观。抓好夜间灯光设计，高标准亮化世纪大桥、南大立交桥、琼州大桥、秀英高架桥，不断提高城市的品位和档次。

四、扩大经济总量，实施优势产业战略，争创产业发展新优势

　　 要在“两地一中心”产业战略的基础上，按照做大做强做精做美的要求，切实做到工业抓总量，旅游抓精品，商贸抓市场，不断增加新的经济增长点。

　　 抓总量，做大优势工业。按照引进项目、培育名牌、形成规模、后发赶超的思路，加大引导、扶持、服务力度，把有产业基础、品牌效应、技术支撑的传统支柱产业，和以电子信息、生物医药、海洋开发、生态环保为主的高新技术产业做大做强，培育一批年产值超10亿元、超100亿元的大中型工业企业。重点扶持以海汽、新大洲为龙头的机械制造业，兴业聚酯为骨干的化纤纺织业，椰树、力神等名牌企业为主的食品饮料加工业，康利、际中等为代表的生物制药业，以及托普、三星等信息产业和海南红塔卷烟厂、椰岛、亚太等烟酒业的发展。加快工业园区资源整合，扩大保税区与国家高新区的政策覆盖面。全面调整规划产业园区布局，着手筹划美安工业区，努力形成狮子岭工业区、桂林洋经济开发区和以南海大道为主轴的现代工业聚集区。今年力争实现工业增加值增长22%，2007年工业总产值和增加值翻一番，把海口建成全省现代工业基地。

　　 抓精品，做旺特色旅游。以“琼州海峡旅游度假区”为重点，沿东海岸和西海岸线建设景观别致的热带风光、滨海特色的观光度假休闲长廊。突出主题公园规划建设，抓好火山口国家地质公园、玉龙泉国家森林公园、红树林特色植物园、好莱坞国际影视城、热带海洋动物园、热带野生动物园、热带植物博览园等重点旅游精品项目的规划建设。加强旅游协作，优化配置旅游资源，发展“海口旅游圈”精品旅游线路，扩大“海口旅游圈”的辐射能力。组建有规模有实力的旅游集团，强化旅游促销，扩大客源市场，促进观光度假、娱乐购物、康体休闲和商务会展旅游的发展。继续旅游市场整顿，强化行业自律，按照国际标准提高旅游业的管理水平和服务质量。力争五年旅游总收入年均增长8%以上。

　　 抓市场，做活商贸业。以建设区域性商贸中心为目标，制定新海口商贸业发展规划，调整优化商品物流业、服务业的布局，千方百计地引进国内外资金雄厚、技术先进、管理水平高、品牌效应好的商贸企业，全面提升我市商贸业的档次和水平。进一步完善火车站、港口、机场的现代物流服务体系，加快发展现代物流业。在城区外围规划建设一批专业交易市场，强化物流集散功能，在城市中心区构建包括海秀路、海府路和老机场在内的中心商业广场，在次中心区建设不同类型的购物商场和门类齐全的购物步行街，形成配套完善、层次分明、布局合理的城市商业服务体系。力争五年社会消费品零售总额年均增长13%以上。

五、以增加农民收入为目标，促进农业全面发展，加快农村小康社会建设

　　“三农”为民生之本，本固则邦宁。全面建设小康社会，重点在农村，难点在农业，焦点在农民。加强“三农”工作是新海口当前一项十分紧迫而重要的任务。我们要抓紧制定新海口的农业发展规划，尽快出台《加快农业发展的若干意见》，加大对农业的投入，大力发展城郊型、加工型、服务型、科技型、生态型农业。2003年农业增加值增长8.5%以上，力争五年内实现农村经济总量翻一番，农业增产、农村增效、农民增收“一翻三增”的目标。

　　 加快农村经济结构调整，发展优势农业。优化农业区域布局，推进优势农产品和特色农产品向优势产区集中，加快发展海洋产业、花卉产业、畜牧业、热带瓜果菜等四大产业，扩大建设无公害蔬菜生产、农副产品加工、农副产品集散批发和良种良苗繁育等四大基地，努力培育水产、花卉、畜牧、乳鸽和瓜菜等五大品牌。积极发展海洋捕捞、海水养殖、海产品加工、海产品营销，培育和打造南海名牌海产品，构建全国水产品电子商务交易平台，把海口建成全省水产品出口生产加工基地和全国热带海产品交易中心。要加强农业标准化建设，加快我市无规定疫病示范区的建设步伐，逐步建立农产品质量安全体系、检测体系、认证体系以及农产品市场准入制度，提高我市农副产品的市场竞争力。要围绕市场抓加工，以龙头企业为引导，全面发展畜禽生产加工业，做大琼山区的乳鸽生产加工产业，做强罗牛山的生猪养殖加工产业。要围绕城市抓调整，走“服务城市、富裕农民”的路子。要围绕需求抓服务，在五大出口处努力构建农产品现代物流服务体系，切实抓好农村剩余劳动力转移，建立多专业、多层次、多形式的服务队伍，为城市发展提供人力资源。

　　 加快推进城镇建设，提高城市化水平。要高标准、高起点地搞好村镇规划，重点推进西秀、红旗、东山、大致坡等镇的城市化改造，形成辐射带动农村经济发展的中心城镇。统筹做好24个镇的道路、供水、供电、供气、广播电视的“五网”规划和建设，搞好农田水利和生态环境等基础设施建设。今明两年要全部解决革命老区和贫困地区人畜饮水困难问题。要消除城乡壁垒，加快解决城中村、城乡结合部的“农转非”问题，缩小农村，减少农民，不断提高城市化水平。

　　 以文明生态村建设为载体，全面提高农村文明程度。文明生态村建设要注重实效，提高质量。要加强农村环境建设，大力整治农村脏乱差，重点清理垃圾杂物，拆除乱搭乱建，实行垃圾定点定期处理，有条件的村庄要建设人厕、猪栏、牛舍“三联通”的沼气池，彻底改变农村与城乡结合部的脏乱差；规划村庄建设，整治村容村貌，硬化亮化绿化美化环境，使农村成为林木环抱、果园飘香、整洁优美、鸟语花香的生活乐园。要提高农民素质，大力实施“十万农民培训计划”，举办各类技术培训班，引导农民学习科学、文化、法律、卫生等知识，指导农民科学合理使用化肥、农药、农膜，增强农民致富奔小康的本领；坚持不懈地组织好文化、科技、卫生、法律下乡活动，传播先进文化，倡导科学文明健康的生活方式。

六、增创体制新优势，提高对外开放水平，增强经济社会发展的动力

　　 创新体制改革。我们必须毫不动摇地坚持多种所有制长期共存，毫不动摇地坚持多种经济成份共同发展，毫不动摇地坚持大力发展非公有制经济。深化国有企业改革。按照建立现代企业制度的要求，继续推进国有企业规范的公司制改革和股份制改造，尚未改制的中小型国有企业要通过改革退出国有序列，大中型国有企业要进一步深化产权改革，政府控股的股份制企业要进一步调整优化国有股比例。继续完善国有资产管理体制，建立权责利统一，管资产和管人、管事相结合的国有资产管理体制，确保国有资产的保值增值。深化财政管理体制改革，构建公共财政框架，优化支出结构，提高财政保障经济发展的能力。要从战略高度放胆放手发展非公有制经济，千方百计地鼓励、支持和引导非公有制经济发展，一心一意地为非公有制经济提供公平公正的法制环境和优质服务，理直气壮地保护非公有制经济的合法权益。要放宽民间资本的市场准入领域，创造市场主体公平竞争的环境。在企业开办、土地使用、信贷扶持、税费优惠、法律服务等方面采取切实可行的措施，全面扶持非公有制经济快速发展。鼓励私营、个体、外资等多种经济成份参与国有企业改组、改造，引导非公有制企业进行股份制或公司制改造，增强非公有制经济的发展能力，全面开拓非公经济发展的新局面。

　　 创新城市经营。经营城市是当今改革的潮流，我们要强化经营城市理念，把城市看作一个资源的集合体，实现城市资源资本化。应用市场机制，把城市资源优势转变为资本优势，把审批资源市场化，公共资源经营化，物化资源货币化，隐形资源显形化，空间资源实物化，全方位经营城市。要完善土地储备制度，垄断一级市场，实行公开拍卖，实现土地配置最优化、价值最大化。要加快推进出租车经营权、户外广告权、道路冠名权等公共资源的公开拍卖，创新城市经营机制。要改革环境卫生、园林绿化等公共事业的管理模式，积极探索社会化、产业化、市场化的经营路子。

　　 创新对外开放。实施开放优先战略，在开放中吸纳人才，在开放中推进产业发展，在开放中创新体制，在开放中形成竞争优势。要创新产业开放。扩大外资投资领域，逐步放开交通运输、商业批发零售、外贸、医疗、教育、旅游和中介服务的外资准入，力争产业开放有新的突破，使海口成为环北部湾地区服务贸易的重要城市之一。要创新招商方式。积极发展高层招商、以商招商、代理招商、定向招商、网上招商等多元化招商，扩大引资规模，提高招商水平。研究制定鼓励跨国公司投资的具体措施，多形式吸引跨国公司到海口投资置业。要创新区域合作。强化中心城市的极化和扩散效应，加强与各市县的经济联系，扩大海口经济腹地，促进优势互补；强化与华南经济圈的经济合作，积极主动地融入其分工合作体系；加强与琼籍华人华侨的联系，充分发挥华侨在招商引资引智中的作用，通过多层次的交流合作，扩大与港澳台及东盟各国的经济合作和贸易往来。

七、创新需求理念，扩大有效需求，不断增强经济发展活力

　　 民富须商活，市强须人旺。必须不断增强投资需求、消费需求、出口需求和就业需求的多重拉动，促进经济快速增长。

扩大投资需求。改革投融资体制，激活社会投资，大规模的引导和吸纳社会资金投向优势产业、城市基础设施、公共事业和社区服务业。继续抓住国家实施积极的财政政策的机遇，组织好环境保护、农田水利、商贸服务业等领域的重大项目申报，争取国债资金和国家开发银行贷款资金支持。优化投资结构，推进百项重点工程计划，筹划和推进一批技术含量高、产业关联度大的新项目，充分发挥项目建设的拉动作用。

　　 搞活消费需求。要制定规划，大力培育健康规范的娱乐业和文化教育产业，形成新的消费需求。要调整布局，建立一批规模大、档次高的专业交易市场，完善连锁店、物流配送中心、电子商务交易等销售体系，激发新的消费需求。要继续落实和调整消费政策，规范整顿市场秩序，改善消费环境，培育新的消费热点。要继续培育假日消费和会展消费，开展多种形式的展销、促销活动，引进外来需求。要培育汽车、住房、电脑等高档消费热点，推进一系列便民措施，促进汽车进入家庭，促进移动通讯、电脑上网和网上购物等信息消费。

　　 增强出口需求。继续用好用足出口扶持政策，营造支持企业出口的良好环境。培育出口龙头企业，建设出口加工基地，实施出口市场多元化战略，开拓新兴出口市场。扩大民营企业外贸出口，优化对外贸易结构，增加深加工、高附加值、高技术含量产品的出口。要培育出口骨干企业，扩大出口货源基地建设，重点建设一批纺织、饮料、机电和生物制药货源基地，增加地产品的出口。要积极实施“走出去”战略，推动优势企业到境外投资、承包工程和劳务合作，不断提高出口对经济增长的贡献率。

　　 扩大就业需求。建立就业担保基金，扩大就业规模，创造更多的就业机会，让更多的城镇居民、大中专毕业生、下岗失业人员尽快实现就业与再就业，提高人民群众收入水平，增强居民的消费能力。继续完善特困居民援助长效机制，逐步解决好人民群众住房难、就医难、入学难、生活难、养老难等问题。

八、大力发展教育产业，弘扬社会主义先进文化，促进各项社会事业全面进步

　　 坚持科教强市战略。科教强市，教育为先。我们必须始终把科技和教育摆在优先发展的战略地位，增强科教创新能力，加快发展教育产业。紧紧依靠科技进步，全面推进技术创新，加速科技成果转化，大力发展高新技术产业，推动大学城、软件园、院士村建设，加快产学研一体化，增强城市发展的动力。继续调整教育布局，巩固提高“两基”成果，加强学校标准建设，优化教师队伍结构，全面推进素质教育，扩大优质初中、高中和职业教育，发展实用技术教育，支持高等教育，把优质教育做大。引导、支持和鼓励社会办教育的积极性，努力形成结构合理化、投资多元化、形式多样化、管理现代化的教育发展新格局，扩大和满足全省以及周边地区对学历教育、优质教育的需求，把海口建成全省的教育消费中心。

　　 加快文化、体育、卫生等社会事业的发展。树立文化是城市战略资源的理念，发掘海瑞、丘浚、冼夫人等历史文化资源，丰富琼剧等地方特色文化，发展具有时代气息的现代文化，建设图书馆、科技馆、歌剧院等文化基础设施，积极发展文化产业。高水平制定娱乐业发展规划，规范管理和积极引导娱乐业，促进娱乐业健康向上有序的发展。加快文化体育中心、足球冬训基地、国奥篮球训练基地等文化体育设施建设，培育文化体育品牌产业。以“低保”为重点，努力建设城乡救助体系，扩大社会保障的覆盖面，切实做好三条保障线的衔接工作。调整优化卫生设施布局，完善社区卫生服务体系和农村卫生保障体系，解决困难群众看病难问题。加强计划生育工作，严格控制人口增长，提高出生人口素质，促进出生人口性别比平衡。努力抓好老干部工作，维护妇女、儿童、老人、残疾人的合法权益。继续做好民政、双拥、外事侨务、民族宗教、对台事务、国防和防震减灾等工作。继续加大社会治安综合治理，建立健全社会治安群防群治网络，严厉打击各种刑事犯罪和经济犯罪，推进安全文明小区、无毒社区建设，加强人民调解工作，维护社会政治稳定。

　　 坚持不懈地开展社会主义精神文明建设。深入开展邓小平理论、“三个代表”重要思想和党的十六大精神的学习活动，唱响主旋律，不断激发全市人民建设小康社会的政治热情。积极实施《公民道德建设实施纲要》，加强社会公德、职业道德、家庭美德教育，努力促进依法治市与以德治市的紧密结合。深入开展文明机关、文明社区、文明诚信企业等群众性创建活动，不断提高城市的文明程度和市民整体素质。

九、优化政务环境，加强政府自身建设，塑造良好的政府形象

　　 建设亲民型政府。善待企业就是善待市民，为社会服务就是为人民服务。各级政府要牢固树立“治政民为重”、“万事民为先”的思想，努力实践“三个代表”的重要思想，认真落实“三个善待”，以人民的利益作为第一需要，以解决人民的困难作为第一选择，以服务人民作为第一责任，以人民是否满意作为第一标准，亲民、为民、便民、利民，不摆花架子，不图虚名，不急于求成，不盲目蛮干，扎扎实实，真抓实干，努力塑造务实创新勤政亲民的政府形象。推进行政管理体制改革，合理设置机构，规范部门职能，优化审批环节，简化办事程序，提高行政效率。强化服务意识，建立政务信息快速处置系统，沟通政府与投资者、政府与市民、政府与游客之间的联系，畅通渠道，解决问题。每个工作人员都要牢记我们是人民政府，没有人民就没有政府，树立公仆意识，始终坚持为人民服务是人民政府的宗旨，做人民公仆是公务员的天职，让百姓高兴和满意是衡量政府工作的根本标准。

　　 建设学习型政府。城市灵魂源于文化，塑造文明重在学习，政府活力贵在学习。按照建设学习型城市的总体要求，建立与时俱进、适合机关工作特点、讲求实效的政府机关学习制度和学习机制。各级政府机关和干部要成为学习的表率，全面推进学习型家庭、学习型社区、学习型社会的建设。学习是为了解放思想，增长知识，提高机关干部驾驭市场经济能力、科学决策能力和依法行政能力。在学习中要坚持正确的、反对错误的、倡导科学的东西，力戒形式主义、力戒教条主义、力戒本本主义。全市机关干部要从旧体制的束缚中解放出来，从旧传统的束缚中解放出来，从旧思想惰性的束缚中解放出来，积极主动运用国内外先进的思想和成果解决海口经济社会发展的实际问题，把思想统一到“两个翻番，两个率先”奋斗目标上来，统一到市委市政府的重大决策上来，推动学习型政府向知识型政府、创新型政府转变。

　　 建设信用型政府。人无信则不立，事无信则不成。没有信用的社会是没有竞争力的社会，没有信用的政府是不可能取信于民的政府。建设信用型政府要把对上负责、对下负责和对民负责有机地统一起来。牢牢树立全市一盘棋的观念，严格行政纪律，确保中央和省以及市委的政令畅通，实现对上的高度负责。全面公开政府机构办事范围、办事程序、办事条件、承诺事项和违诺责任等相关行政事项，实现对下的高度负责，取信于企。在制定全市经济发展的重大决策、城市建设的重大项目、“三农”问题的重大意见、重大政策法规和为民办实事事项时，都必须以人民的根本利益为依据，努力做到言必信、行必果，实现对市民的高度负责，取信于民。强化行政信用监督，突出人大依法监督、政协民主监督、机关内部监督、服务对象监督、人民群众监督和社会舆论监督。加强机关及工作人员失信违约责任追究的制度建设，建立行政告诫制度，增强行政效能考核的刚性约束，以信用政府推动信用海口建设。

　　 建设清廉型政府。清则无惧，廉则生威。各级政府和工作人员要牢记“两个务必”，倡导讲实话、使实劲、办实事、求实效，反对官僚主义和形式主义；提倡艰苦奋斗、埋头苦干、密切联系群众，反对奢侈浪费、热衷应酬、脱离群众的作风。实施“阳光政务”，推广电子政务，继续完善“三制”，充分发挥重点项目服务中心和投资环境监督中心的作用，全面推行首问制、导向制和责任追究制，提高行政透明度。认真开展行政重大事项听证制度，推进政府决策的民主化、公开化、程序化、规范化、科学化。继续执行党风廉政建设责任制，推进党风廉政建设和反腐败工作。加强勤政廉政教育，努力做到堂堂正正做官，清清白白做事，兢兢业业为民。坚决严格查处各类大案要案，坚决严厉惩处腐败分子；坚决杜绝“吃、拿、卡、要”现象，坚决处理违法违纪人员。以廉政勤政的实际成果，赢得广大人民群众的拥护和信赖。

　　 各位代表！全面建设小康海口的伟大工程已经启动，我们要解放思想，开拓前进。齐心协力博今日，引吭高歌迎明天。搏今日，我们千难万险都不怕，志同道合，励精图治。迎明天，我们欢声笑语，携手共进，谱写海口光辉灿烂的未来。让我们在以胡锦涛同志为总书记的党中央的领导下，高举邓小平理论伟大旗帜，全面贯彻“三个代表”重要思想，与时俱进，开拓进取，扎实工作，为全面建设小康社会，率先基本实现现代化的目标而努力奋斗！
